# Cytokine and chemokine profiles in fibromyalgia, rheumatoid arthritis and systemic lupus erythematosus: a potentially useful tool in differential diagnosis

**DOI:** 10.1007/s00296-014-3172-2

**Published:** 2014-11-07

**Authors:** Daniel J. Wallace, Igor M. Gavin, Oleksly Karpenko, Farnaz Barkhordar, Bruce S. Gillis

**Affiliations:** 1 Wallace Rheumatic Diseases Study Center, West Hollywood, CA USA; 2 EpicGenetics, Los Angeles, CA USA; 3 Research Resources Center, University of Illinois at Chicago, Chicago, IL USA; 4 College of Medicine, University of Illinois at Chicago, Chicago, IL USA

**Keywords:** Fibromyalgia, Rheumatoid arthritis, Systemic lupus erythematosus, Cytokines, Chemokines, Peripheral blood mononuclear cells, Phytohemagglutinin

## Abstract

Making a correct diagnosis is pivotal in the practice of clinical rheumatology. Occasionally, the consultation fails to provide desired clarity in making labeling an individual as having fibromyalgia (FM), systemic lupus erythematosus (SLE) or rheumatoid arthritis (RA). A chemokine and cytokine multiplex assay was developed and tested with the goal of improving and achieving an accurate differential diagnosis. 160 patients with FM, 98 with RA and 100 with SLE fulfilling accepted criteria were recruited and compared to 119 controls. Supernatant cytokine concentrations for IL-6, IL-8, MIP-1 alpha and MIP-1 beta were determined using the Luminex multiplex immunoassay bead array technology after mitogenic stimulation of cultured peripheral blood mononuclear cells. Each patient’s profile was scored using a logistical regression model to achieve statistically determined weighting for each chemokine and cytokine. Among the 477 patients evaluated, the mean scores for FM (1.7 ± 1.2; 1.52–1.89), controls (−3.56 ± 5.7; −4.59 to −2.54), RA (−0.68 ± 2.26; −1.12 to −0.23) and SLE (−1.45 ± 3.34, −2.1 to −0.79). Ninety-three percent with FM scored positive compared to only 11 % of healthy controls, 69 % RA or 71 % SLE patients had negative scores. The sensitivity, specificity, positive predictive and negative predictive value for having FM compared to controls was 93, 89, 92 and 91 %, respectively (*p* < 2.2 × 10^−16^). Evaluating cytokine and chemokine profiles in stimulated cells reveals patterns that are uniquely present in patients with FM. This assay can be a useful tool in assisting clinicians in differentiating systemic inflammatory autoimmune processes from FM and its related syndromes and healthy individuals.

## Introduction

The differential diagnosis of rheumatic disorders involves distinguishing inflammation, degenerative changes and central sensitization from each other. Making this distinction is usually obvious or easily discernible on the basis of taking a history, performing a physical examination, viewing imaging studies as appropriate and obtaining laboratory analyses. There are occasions where traditional methods fail to either elicit a diagnosis or give the clinician clarity with regards to whether a patient, for example, is inflamed or has fibromyalgia (FM) [1].

Fibromyalgia has been a medical condition that for decades was relegated to being defined as a collection of subjective symptoms of varying characteristics lacking objective and reproducible laboratory features [2]. FM is estimated to afflict a population in the USA alone of between 2 and 5 % of the US population [3]. The annual cost of diagnosing FM makes it one of the most expensive diagnostic processes linked to any illness [4] as it presently chiefly relies on using tests to “rule out” disorders of rheumatic, neurologic, psychiatric, hematologic and endocrine origins. Hence, finding an objective diagnostic methodology offers substantial medical cost benefits. It additionally could lead to a potential way to monitor treatment efficacy as well as eliminate what has been an inexorable and prolonged pathway to an endpoint diagnosis that has previously utilized an indirect process of elimination.

In the last few years, laboratory panels have demonstrated that the degree or intensity of inflammation, for instance, may be further elucidated by composite scores or weighted testing that are statistically significant (e.g., multibiomarker disease activity <Vectra> for RA, cell-bound complement activation products <Avise> for SLE) [5, 6]. The multibiomarker disease activity index used in RA, for example, includes cytokine levels in its weighted scoring. In 2012, a group at the University of Illinois College of Medicine evaluated 8 cytokines and chemokines in 110 FM patients and 91 matched healthy controls [7]. Unlike most previous studies, rather than measuring serum levels, peripheral blood mononuclear cells (PBMC) were stimulated by mitogens and in vitro production was quantitated. Statistically, significant findings distinguished the two groups as the FM group had 1.4-8 fold decreases in cytokine/chemokine production. Post hoc analyses determined that the panel was just as accurate if four chemokines and cytokines (instead of eight) were studied. Since FM is common in patients with autoimmune disorders, such as systemic lupus erythematosus (SLE) [8, 9], Sjogren’s Syndrome [10, 11] and rheumatoid arthritis (RA) [12], the chemokine/cytokine patterns found in FM patients may not have been unique to FM and were perhaps merely linked to rheumatic disorders as a whole, especially because of the frequent coexistence of FM and RA and FM and SLE. We therefore undertook comparative analyses to test the hypothesis that inflammation (represented by RA and SLE) and central sensitization syndromes (represented by FM) have distinct cytokine/chemokine signatures that could be clinically relevant.

## Methods

A total of 160 FM patients diagnosed with FM for at least 1 year were recruited to the study. Patients underwent two separate and independent physical examinations by two physicians to confirm that they met the American College of Rheumatology criteria [2]. All FM patients had discontinued their FM-related medications for 2 weeks prior to testing. One hundred SLE and 98 RA patients treated in University of Illinois College of Medicine were also studied. All fulfilled American College of Rheumatology criteria [13, 14]. Information regarding their demographics, disease duration and concurrent medication was collected (Table 1). Patients with SLE or RA who also had FM were excluded. The healthy controls were age- and sex-matched to the FM patients only and lacked a history of any type of chronic or acute illnesses and none were using any medications, over the counter or prescription drugs that had any known immunologic effects. After obtaining IRB approved informed consent, PBMC were obtained from each patient as described earlier [7]. Concentrations of live cells as well as percentages of dead cells in PBMC fractions were measured by using an automatic cell counter (Cellometer Auto 2000, Naxcelom Biosciences) according to the manufacturer’s instructions. These cells were cultured in the presence of the mitogenic activator phytohemagglutinin (PHA). After a culture time of 24 h, the resultant supernatant was harvested. Supernatant cytokine concentrations of interleukin-6, interleukin-8, macrophage inflammatory protein-1 alpha and macrophage inflammatory protein-beta (IL-6, IL-8, MIP-1a and MIP-1b) were determined using the Luminex multiplex immunoassay bead array technology [7]. Serial dilutions of cytokine standards were run in duplicates in each assay; their readings were used for calculating standard curves. In addition, pooled culture supernatants obtained from activated cell cultures served as a positive control. Fluorescence was measured using a MagPix fluorescence bead reader (Luminex).

**Table 1:** Characteristics of patients

	Control	FM	RA	SLE
Cohort size	119	160	98	100
Age				
Range	18–76	18–82	21–92	21–92
Median	41	53.2	59	48
Mean	40.8	52.2	58.1	49.6
Sex				
Female	101	98	86	94
Male	18	12	12	6

### Statistical analyses

Fluorescence intensities were transferred into R (http:www.r-project.org) statistical software [15] for converting into concentration values. The standard curve was fitted with a 5-PL model, and the concentrations of the cytokines were quantified according to the curve. A two-sided *t* test with unequal variance was used to test whether the mean concentrations of each cytokine were the same in the groups. The descriptive statistics of the groups as well as the *p* values of the *t* test were calculated by using the stats package for R.

We used function lrm* from package rms [16] in R software to determine a logistical regression model on the concentration of the cytokines described above as predictor variables, and binary dependent variables and test scores were calculated.

The measured concentrations of the four cytokines, *c*_IL-6_, *c*_IL-8_, *c*_MIP-1a and_ *c*_MIP-1b_, are combined into a single score using the following formula: *x* = intercept + *w*_IL-6_ *c*_IL-6_ + *w*_IL-8_ *c*_IL-8_ + *w*_MIP-1a_ *c*_MIP-1a_ + *w*_MIP-1b_ *c*_MIP-1b_. Weights *w*_IL-6_, *w*_IL-8_, *w*_MIP-1a_ and *w*_MIP-1b_, and intercept were determined experimentally by fitting a logistic regression curve. In addition, a cytokine/chemokine composite test score on the scale of 0–100 was calculated using the following formula:

$$ {\text{Cytokine/chemokine composite test score}} = \frac{1}{{1 + e^{ - x} }}*100 \, . $$

## Results

**Fig. 1:**
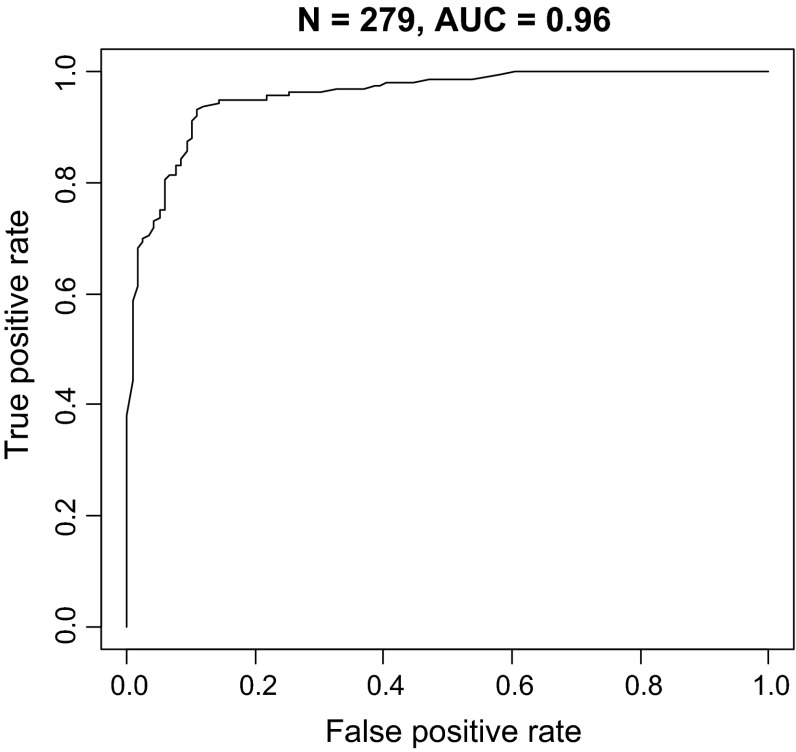
Receiver operating characteristic (ROC) curve for CCC test scores of 160 FM patients and 119 controls. The area under the curve (AUC) is 0.96

Fibromyalgia patients clearly had higher scores; the mean score was 1.7 ± 1.2, with 1.52–1.89 confidence interval which corresponds to 84.6 value of cytokine/chemokine test score on the scale of 0–100 (Table 2). The mean score of the control patients was −3.56 ± 5.7, with −4.59 to −2.54 confidence interval (cytokine/chemokine composite <CCC> test score 2.8). If a “profile” meant having a positive score (or greater than 50 on a scale of 100 for CCC test score), 93 % with FM and only 11 % of healthy controls met this benchmark. This was considered to be 93 % sensitive and 89.4 % specific for the diagnosis of FM when compared to the 119 controls (Table 3). The Wilcoxon test for FM versus healthy controls was *p* < 2.2 × 10^−16^, and the area under the receiver operating characteristic (ROC) curve was 0.96 (Fig. 1). Following completion of this part of the protocol, 100 patients with SLE and 98 RA patients were studied. A total of 71 % of those with SLE and 69 % with RA had negative scores (<50 on a scale of 100). The mean scores were −0.68 ± 2.26 for RA patients (−1.12 to −0.23 confidence interval) and −1.45 ± 3.34 for SLE patients (−2.1 to −0.79 confidence interval). Corresponding cytokine/chemokine composite test scores were 33.7 and 19 on a scale of 100.

**Table 2:** Cytokine/chemokine composite (CCC) test scores of patients and healthy controls

	Subjects tested	Mean score	95 % Confidence interval	CCC test score	% Positive	% Negative
Healthy controls	119	−3.56 ± 5.7	−4.59 to −2.54	2.8	13 (11 %)	106 (89 %)
FM	160	1.7 ± 1.2	1.52 to 1.89	84.6	149 (93 %)	11 (7 %)
RA	98	−0.68 ± 2.26	−1.12 to −0.23	33.7	30 (31 %)	68 (69 %)
SLE	100	1.45 ± 3.34	−2.1 to −0.79	19	29 (29 %)	71 (71 %)

**Table 3:** Characteristics of the CCC test scores in FM versus controls

Sensitivity	93 %
Specificity	89 %
Positive predictive value	92 %
Negative predictive value	91 %
Area under ROC curve	0.96
Wilcoxon test for FM scores versus controls	*p* < 2.2 × 10^−16^

**Fig. 2:**
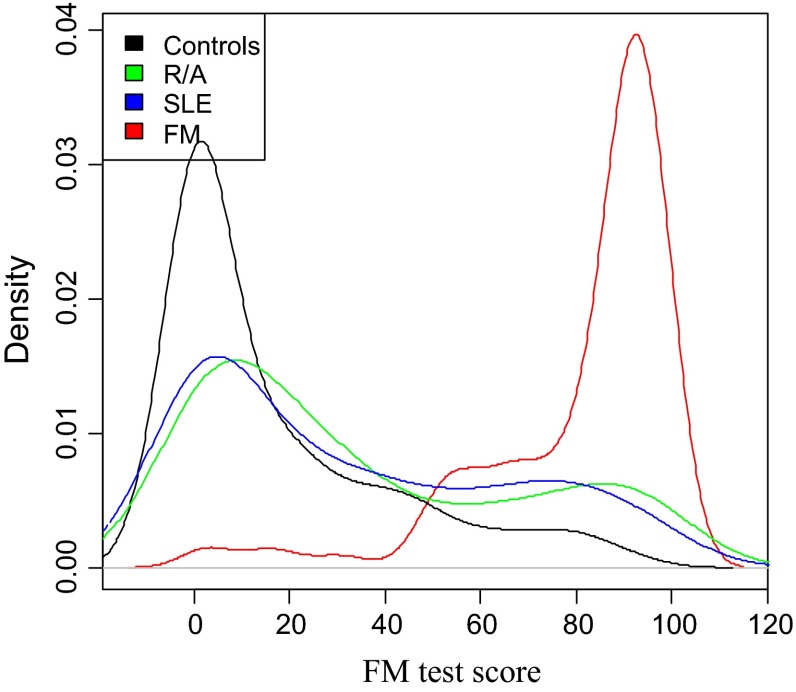
The probability densities of CCC test scores of 119 controls, 160 FM patients, 100 SLE patients and 98 RA patients

To better understand the statistical distribution of cytokine profile scores, each patient’s test score was calculated as a percentile rounded to the nearest integer on a scale of 1–100. There were two peaks in the scoring distribution of the RA and SLE patients: one in the low score region (48 % with SLE and 42 % with RA for example had scores between 0 and 29) and a second smaller peak in the highest scoring region (25 % with SLE and 28 % with RA had scores between 70 and 100) (Fig. 2). This suggests that immune modulating agents, corticosteroids or concurrent FM in the autoimmune patients may account for the findings. Nevertheless, the SLE/RA versus FM scores achieved a significance of *p* < 2.2 × 10^−16^ as calculated by the Wilcoxon rank sum test. Concentrations of the studied cytokines in PBMC culture supernatants tended to be lower in FM patients compared to the autoimmune subjects, except for RA where IL-8 concentrations were similar (Table 4). Interestingly, FM patients showed the lowest levels of IL-6 compared to RA, SLE patients and controls—which were within 2 % of each other (*p* < 0.00001). Most of the autoimmune patients disease was under good control with medication.

**Table 4:** Wilcoxon rank sum test of differences in stimulated cytokine/chemokine levels in SLE and RA samples compared to either FM samples or controls

Cytokine	Controls			FM			SLE					RA				
	Mean	Med.	SD	Mean	Med.	SD	Mean	Med.	SD	Contr. FDR	FM FDR	Mean	Med.	SD	Contr. FDR	FM FDR
IL.6	5,365	4,895	1,901	2,667	2,543	1,149	5,012	4,554	2,704	0.62	<0.00001	4,560	4,279	2,044	0.08	<0.00001
IL.8	24,009	24,126	10,433	17,298	16,592	7,094	20,102	15,359	13,666	0.44	0.10	16,387	12,487	11,848	0.01	0.56
MIP-1a	1,876	1,723	777	1,104	1,085	465	1,902	1,678	1,078	0.88	<0.00001	1,553	1,465	801	0.08	0.00004
MIP-1b	18,521	16,725	7,814	10,756	10,556	4,350	19,929	16,719	12,430	0.62	<0.00001	15,583	13,985	7,735	0.09	<0.00001

*p* values were adjusted for multiple comparisons by using Benjamini–Hochberg procedure

## Discussion

Nearly 500 FM, autoimmune and healthy control patients underwent testing for cytokine/chemokine activity after mitogenic stimulation. Using a numerical score, all three groups had unique patterns with FM patients demonstrating less response to stimulation. This cytokine profile test had a 93 % sensitivity and an 89.4 % specificity for the diagnosis of FM. We also found that these profiles are relatively sensitive and specific for FM compared to SLE and RA. It remains unclear if these differences are directly related to the pathogenesis of FM.

We have been studying the role of cytokines in fibromyalgia for 30 years [17, 18]. Tumor necrosis factor and interleukins 1, 2, 6, 8 and 10 are associated with pain modulation, sleep induction, cognitive dysfunction, antinociception and sympathetic nervous system homeostasis to varying degrees. Studies assessing serum levels in our opinion are unreliable due to short half-lives, circadian rhythms (time of day that the measurements are obtained) as well as target tissue variance. Published reports have not demonstrated a consistent FM pattern. A few studies have looked at cytokine function after mitogenic stimulation. Patients with self-reported FM of <2 years duration had a greater response to stimulation than those with chronic FM [17]. This is consistent with findings from the National Institutes of Health that although serum cortisol levels are normal in the syndrome, response to cortrosyn stimulation is blunted [18]. Further work has suggested that sympathetic nervous system responses are less robust in FM when ascertained by decreased heart rate variability or exercise [19, 20].

The scores found in this report are consistent with the finding that chronic stress decreases cytokine/chemokine responses. IL-6 is an acute phase reactant that is associated with stress, fatigue, hyperalgesia and sympathetic nervous system activation; IL-8 induces chemotaxis, phagocytosis, angiogenesis and modulates sympathetic mediated pain, and MIP-1 alpha and beta recruit polymorphonuclear cells and are chemoattractants for natural killer cells and monocytes. In all probability, chronic sensitization syndromes (which includes FM) are associated with a milieu whereby responses to sympathetic, hormonal, cytokine and chemokine stimulation are diminished. In the last decade, it has also become evident that glial cells produce cytokines, and complex interactions in the setting of FM might explain opioid induced hyperalgesia observed in the syndrome [21]. In other words, FM patients often fare worse when prescribed narcotic analgesics.

The expression of serum cytokines and chemokines in RA and SLE is increased, and the use of anticytokine therapies (and Phase 1 and 2 studies of chemokine inhibition) for autoimmune disease suggests that the findings would be opposite of what has been shown in FM [22].

Our study was confounded by different times of day that the cytokine/chemokine profile was obtained, concomitant anti-inflammatory and immune modulating medications, the use of corticosteroids and coexistence of FM in <10 % of the autoimmune (SLE and RA) patients. Despite this, we were able to demonstrate statistically significant differences in scores comparing patients with FM, healthy controls and autoimmune disease. The study was exploratory and provided preliminary information documenting a signal. Now that there is one, further data mining and/or a more focused second study would allow us to examine which subsets or autoimmune “phenotypes” are more likely to influence a combined chemokine score. This would, for example, include looking at established disease activity indices and acute phase reactants (e.g., sedimentation rate, C reactive protein). High cytokine/chemokine composite test scores strongly suggest a central sensitization component with or without an inflammatory condition. We posit that using a cytokine/chemokine stimulated response composite score is clinically useful in the differential diagnosis of FM patients as well as in patients where the role of inflammation versus central sensitization would benefit from further delineation. For example, changes in the score could be used to monitor response to different interventions.
